# Surface-Charge Characterization of Nanocomposite Cellulose Acetate/Silver Membranes and BSA Permeation Performance

**DOI:** 10.3390/membranes15020061

**Published:** 2025-02-11

**Authors:** Ana Sofia Figueiredo, María Guadalupe Sánchez-Loredo, Maria Norberta de Pinho, Miguel Minhalma

**Affiliations:** 1 Department of Chemical Engineering, Instituto Superior de Engenharia de Lisboa, Polytechnic University of Lisbon, Rua Conselheiro Emídio Navarro 1, 1959-007 Lisbon, Portugal; ana.figueiredo@isel.pt; 2 CeFEMA, Department of Chemical Engineering, Instituto Superior Técnico, Universidade de Lisboa, Av. Rovisco Pais, 1, 1049-001 Lisbon, Portugal; marianpinho@tecnico.ulisboa.pt; 3 Instituto de Metalurgia, Facultad de Ingeniería, Universidad Autónoma de San Luis Potosí, Sierra Leona 550, San Luis Potosí 78210, Mexico; msanchez@uaslp.mx

**Keywords:** cellulose acetate membranes, silver nanocomposite membranes, streaming potential, zeta potential, BSA permeation, ultrafiltration

## Abstract

Membrane processes are a reality in a wide range of industrial applications, and efforts to continuously enhance their performance are being pursued. The major drawbacks encountered are related to the minimization of polarization concentration, fouling, and biofouling formation. In this study, silver nanoparticles were added to the casting solutions of cellulose acetate membranes in order to obtain new hybrid membranes that present characteristics inherent to the silver nanoparticles, namely antibacterial behavior that leads to biofouling reduction. A systematic study was developed to assess the effect of ionic strength, membrane polymeric structure, and silver nanoparticle incorporation on the cellulose acetate (CA) membrane surface charge. Surface charge was quantified by streaming potential measurements and it was correlated with BSA permeation performance. CA membranes were prepared by the phase-inversion method using three casting-solution compositions, to obtain membranes with different polymeric structures (CA400-22, CA400-30, CA400-34). The nanocomposite CA/silver membranes (CA/Ag) were prepared through the incorporation of silver nanoparticles (0.1 and 0.4 wt% Ag) in the casting solutions of the membranes. To evaluate the electrolyte concentration effect on the membranes zeta potential and surface charge, two potassium chloride solutions of 1 mM and 5 mM were used, in the pH range between 4 and 9. The results show that the zeta-potential values of CA/Ag membranes were less negative when compared to the silver-free membranes, and almost independent of the silver content and the pH of the solution. The influence of the protein solution pH and the protein charge in the BSA solutions permeation was studied. The pH conditions that led to the lower permeate fluxes were observed at the isoelectric point of BSA, pH = 4.8.

## 1. Introduction

Membrane processes are increasingly used in industrial applications, with advantages over other unit operations including lower power requirements, no need for additives, no phase change, ease in scaling up, and integration into other separation processes. Nevertheless, the pressure-driven membrane processes are still often associated with problems of concentration polarization, membrane fouling and biofouling, usually originating from the adsorption of organic substances on the membrane surface. This adsorption could affect the membrane hydrophilicity, the surface charge, and the pore size, which may lead to changes in the membrane transport properties [1,2,3,4,5,6].

Some researchers have studied the fabrication of polymeric membranes with the addition of nanoparticles such as silver (Ag), silica (SiO_2_), zirconia (ZrO_2_), zinc oxide (ZnO), and titania (TiO_2_) to obtain membranes with enhanced properties in terms of hydrophilicity, permeability, antifouling, solute rejection, and increased lifetime [1,5,7,8,9,10,11,12,13,14,15].

Since ancient times, silver has been used to control the putrefaction of liquids and as a mitigation agent to the incursion and spread of diseases, due to its extraordinary antibacterial properties [7,16]. Nowadays, it is recognized that even a low concentration of silver nanoparticles (AgNPs) provides antibacterial effects, and therefore, there has been an increase in research studies that consider the incorporation of silver nanoparticles in polymeric membranes [7,8,9,10,11,17,18].

In addition to the antibacterial properties revealed by the polymeric membranes with silver nanoparticles, studies reported that the incorporation of silver nanoparticles conferred improved characteristics in terms of hydrophilicity and antifouling to the nanocomposite membranes [10,19,20]. Zodrow et al. prepared polysulfone ultrafiltration membranes impregnated with silver nanoparticles and observed very similar results in terms of permeability and surface charge for membranes with 0 and 0.9% (by weight) of silver nanoparticles and an enhancement in terms of hydrophilicity for the silver-containing membranes [19]. Another study, developed by Li et al., shows that the incorporation of silver nanoparticles onto poly(vinylidene fluoride) (PVDF) membrane improved the surface hydrophilicity and the anti-organic fouling performance of the modified membrane [10]. Another study on polyethersulfone (PES) membranes with embedded silver nanoparticles revealed that the contact angles of the blended membranes gradually declined when the amount of silver nanoparticles increased in the mixed matrix membranes, indicating that the hydrophilicity of the PES membranes increased in the presence of silver nanoparticles [20].

Traditional membrane characterization parameters, such as molecular weight cut-off value and pore-size distribution, are currently being supplemented by membrane surface characteristics, such as surface-charge density or zeta potential [21]. Most membranes, when brought into contact with an aqueous solution, acquire an electric surface charge through several possible mechanisms. These mechanisms may include the dissociation of functional groups, adsorption of ions from solution, and adsorption of polyelectrolytes, ionic surfactants, and charged macromolecules. These surface charges have an influence on the distribution of ions in the solution due to the requirement of the electroneutrality of the system. This leads to the formation of an electrical double layer, so that a charged surface and a neutralizing excess of counter-ions are present in the adjacent solution [22]. It is recognized that the charge or electrical potential properties of membranes have a very substantial influence on their filtration performances. That is the reason why there is a great interest in characterizing these properties. The streaming potential technique is used to determine the zeta potential of membranes, and the streaming potential occurs when there is a relative motion between a fluid containing charged species and a charged surface, caused by a hydrostatic pressure gradient. In the case of asymmetric or composite plane membranes, the zeta potential is now frequently determined from tangential-flow streaming potential measurements [23]. Some researchers use streaming potential measurements in their studies, e.g., Nyström et al. [24]. They studied the rate and extent of protein adsorption and fouling during ultrafiltration through the monitoring of changes in the membrane charge, evaluated by streaming potential data; also, Nabe et al. used the same technique to evaluate membrane-surface modifications [25].

Besides complying with the important requisite of large-scale manufacturing, the cellulose acetate (CA) membranes prepared by the phase-inversion technique offer multiple possibilities for developing novel materials as membrane/nanoparticles composites with a lesser tendency for fouling. In the present work, cellulose acetate membranes were prepared with different ratios of acetone/formamide with 0 wt% Ag (CA400-22, CA400-30, CA400-34), 0.1 wt% Ag (CA400-22Ag0.1, CA400-30Ag0.1, CA400-34Ag0.1), and 0.4 wt% Ag (CA400-22Ag0.4, CA400-30Ag0.4, CA400-34Ag0.4). For these membranes, experiments envisaging the study of the influence of solution ionic strength, solution pH, membrane polymeric structure, and the incorporation of silver nanoparticles on the surface zeta potential of cellulose acetate membranes were carried out. The zeta potential was calculated using data from streaming potential measurements. The influence of both membrane charge and pH on the permeation of BSA solutions was also assessed.

## 2. Materials and Methods

### 2.1. Materials

Cellulose acetate with an average molecular weight of 30,000 g/mol and a degree of substitution of 39.8% acetyl groups (Sigma-Aldrich, Darmstadt, Germany) was used as the membrane-forming polymer. Acetone (Labchem, Zelienople, PA, USA) and formamide (Sigma-Aldrich, Darmstadt, Germany) were used as such without further purification, as solvent and co-solvent, respectively. Silver nitrate (Panreac, Barcelona, Spain), polyvinylpyrrolidone (PVP) (BDH Chemicals, Dubai, United Arab Emirates), and sodium borohydride (Panreac, Barcelona, Spain) were used in the synthesis of silver nanoparticles, without additional purification. The electrolyte solutions for the zeta potential experiments were prepared with potassium chloride (Merck-Schuchardt, Munich, Germany), and the solutions to adjust the pH were obtained from hydrochloric acid 37% (Panreac, Barcelona, Spain) and sodium hydroxide pellets (Panreac, Barcelona, Spain). The aqueous solutions were all prepared with deionized water.

### 2.2. Membrane Preparation

CA and CA/Ag flat-sheet membranes were prepared by the wet-phase-inversion method described by Kunst and Sourirajan [26]. Table 1 shows the casting solutions’ compositions and the casting conditions of the prepared membranes. The silver nanoparticles were prepared according to the authors’ previous work [15]. For nanoparticle incorporation, the modified casting solution was prepared by the addition of the dispersion of Ag nanoparticles to acetone (Table 1). Membranes are identified in Table 1 where “22, 30, and 34” represent the concentration of formamide, wt%, considering the CA400 series free of silver (i.e., CA400-22, CA400-30, and CA400-34). For CA/Ag nanocomposite membranes, the symbols “Ag0.1 and Ag0.4” represent the silver content of 0.1 wt% Ag and 0.4 wt% Ag, respectively (i.e., CA400-30Ag0.1 and CA400-30Ag0.4).

### 2.3. Pure-Water Permeation Flux and Hydraulic Permeability

The permeation experiments were carried out with deionized water, to determine the membrane pure-water flux, J_pw_. The pure-water permeation flux was determined by weighing the permeate collected (M_p_) in a given time (t) and dividing it by the membrane area (A_m_) as J_pw_ = M_p_/(A_m_∙t).

The hydraulic permeability (L_p_) is obtained by the slope of the linear variation between the pure water permeate flux (J_pw_) as a function of the transmembrane pressure (ΔP), J_pw_ = L_p_ × ΔP. The range of the transmembrane pressure was 1, 2, and 3 bar with a feed flowrate of 180 L/h. The permeation experiments were performed in an installation consisting of a feed tank, a pump, a flowmeter, five permeation cells, a valve, and two manometers (placed before and after the permeation cells).

The system pressure and the feed flowrate are assured by the pump, and the pressure is adjusted by the valve. The five permeation cells are flat-plate cells with two detachable parts separated by a porous plate (membrane support) with a membrane surface area of 13.2 × 10^−4^ m^2^ described previously by Afonso and de Pinho [27]. Before the experiments, membranes were compacted for 2 h with deionized water at a transmembrane pressure of 3 bar, and the stabilization time for each experiment run was 30 min.

### 2.4. Membrane Molecular Weight Cut-Off

The molecular weight cut-off (MWCO) parameter, defined by the molecular weight of a determined macromolecule whose rejection is approximately 91%, was determined using the apparent rejections coefficient of reference neutral organic solutes, using several polyethylene glycol (PEG) and dextran solutions (PEG 1000, 3000, 6000, 10,000 and 20,000 Da; Dextran 40,000 Da).

The apparent solute rejection coefficient (f) is defined as f = (C_f_ − C_p_)/C_f_, where C_f_ and C_p_ are the solute concentrations in the bulk of the feed solution and in the permeate solution, respectively. To determine the MWCO, the straight line of log(f/(1−f)) as a function of the molecular weight of the organic solutes was used. The MWCO value is obtained by the intersection of that straight line with the value 1, which corresponds to a value of apparent rejection coefficient of 90.9%.

The permeation of organic solutes was performed using solutions with 600 ppm at 180 L/h and 1 bar; the stabilization time for each experimental run was 30 min. The organic solutes’ concentration in the feed and in the permeate solutions were determined in terms of total organic carbon (TOC) content, using a Dohrmann Total Organic Carbon Analyzer Model DC-85A.

### 2.5. Streaming Potential Measurements

The CA and CA/Ag membranes zeta potential was determined from streaming potential measurements using an EKA electrokinetic analyzer (Anton Paar, Graz, Austria) equipped with a rectangular cell. Two pieces of membranes, with dimensions 125 by 50 mm, were mounted in the measuring cell with the active layer facing each other. Four polytetrafluoroethylene (PTFE) spacers were placed between the membranes, each one with a thickness of 0.254 mm and a longitudinal slit creating a channel between the two membranes. The streaming potentials were measured along the surface of clean membranes as a function of pH and electrolyte concentration. The electrolyte solution used in this experimental work was potassium chloride with different ionic strengths, ranging from 1 mM to 5 mM. The streaming potential measurements were performed with an applied pressure of 0.5 bar at the pH range of 4–9. The pH was set within the range by adding small amounts of NaOH 0.1 M and HCl 0.1 M.

The zeta potential (*ζ*) was calculated by the Helmholtz–Smoluchowski equation, Equation (1), where *U_S_* is the streaming potential, Δ*P* is the applied differential pressure, *µ* the viscosity, ε_0_ the permittivity of vacuum, *ε* the permittivity of the test solution, *L* the channel length, *A* the cross-sectional area, and *R* is the channel resistance [28,29].

(1) $$\zeta =\frac{U_{S}}{\Delta P}\times \frac{\mu }{\varepsilon \times \varepsilon_{0}}\times \frac{L}{A}\times \frac{1}{R}$$

Using the Fairbrother and Mastin approach, the ratio *L*/*A* can be considered as expressed in Equation (2), where *k*_0.1_ and *R*_0.1_ are the conductivity and the resistance of the channel with a 0.1 M potassium chloride solution. Due to the high electrical conductivity of the electrolyte solution, the surface conductivity may be neglected, obtaining correct values for the ratio *L*/*A*. At the end of each zeta-potential measurement, the cell is filled with a 0.1 M KCl solution in order to measure the conductivity and the cell resistance [30,31].

(2) $$\frac{L}{A}=k_{0.1}\times R_{0.1}$$

Replacing Equation (2) in Equation (1), the final expression used to calculate the membrane zeta potential is obtained, Equation (3).

(3) $$\zeta =\frac{U_{S}}{\Delta P}\times \frac{\mu }{\varepsilon \times \varepsilon_{0}}\times \frac{1}{R}\times k_{0.1}\times R_{0.1}$$

The Debye length can be determined using the relation presented in Equation (4) [32]:

(4) $$k^{-1}={\left(\frac{\varepsilon_{0}\times \varepsilon \times k_{B}\times T}{2\times Z^{2}\times e^{2}\times N_{A}\times C_{S}}\right)}^{1/2}$$

where *k_B_* is the Boltzmann constant, *T* is absolute temperature, *Z* is the ion valence, *e* is the fundamental electron charge, *N_A_* the Avogadro’s number, and *C_S_* is the electrolyte concentration.

Even though zeta potential is the most commonly used electrokinetic parameter to characterize the interface electrolyte/membrane, the surface-charge density at shear plane can also be used and it is determined by the Gouy–Chapmann equation, Equation (5) [33,34]:

(5) $$\sigma_{e}=\frac{2\times \varepsilon_{0}\times \varepsilon \times k\times k_{B}\times T}{Z\times e}\times sinh\left(\frac{Z\times e\times \zeta }{2\times k_{B}\times T}\right)$$

where *κ* is the inverse of Debye length and *ζ* is the zeta potential.

### 2.6. Bovine-Serum-Albumin Permeation Experiments

Permeation experiments were carried out with Bovine Serum Albumin (BSA) (Sigma-Aldrich, Darmstadt, Germany, lyophilized powder, crystallized, ≥98.0%) solutions of 50 ppm at three different pH values (below the protein isoelectric point (pH 4), at isoelectric point (pH 4.8) and above the BSA isoelectric point (pH 6)). The BSA solutions were prepared in 10 mM buffer solutions according to the pH value established for the solutions; thus, for the solutions at pH 4 and 4.8, a citric buffer was used; for pH 6.0, a phosphate buffer was prepared. The permeation experiments were conducted with a feed flowrate of 180 L/h and with a transmembrane pressure of 1, 2, and 3 bar. Permeate samples were taken every 30 min to evaluate the permeate flux variation during the permeation time.

## 3. Results and Discussion

### 3.1. Pure-Water Flux Permeation and Hydraulic Permeability

**Figure 1:**
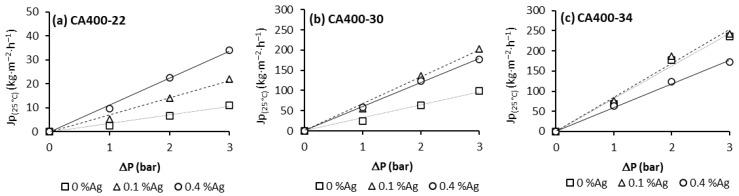
Pure water flux obtained at 180 L/h and transmembrane pressure of 1, 2 and 3 bar for CA and CA/Ag membrane series: (**a**) CA400-22, (**b**) CA400-30 and (**c**) CA400-34.

Figure 1 summarizes the pure-water flux permeation (J_p_) results. For these experiments, CA membranes and CA/Ag membranes from different casting solutions and different amounts of silver nanoparticle content were used [18].

From Figure 1, it is possible to observe that, for all membranes, the pure-water flux increases with the transmembrane pressure in a linear relation; the hydraulic permeability results are presented in Table 2.

Comparing the hydraulic permeability results for membranes obtained from casting solution CA400-22, an increase in L_p_ with the silver nanoparticles content is noted. For casting solutions CA400-30 and CA400-34, the enhancement of hydraulic permeability values is observed with the incorporation of 0.1 wt% Ag, but with the incorporation of 0.4 wt% Ag nanoparticles, this enhancement was not observed. The membrane CA400-34Ag0.4 presents a decrease in the hydraulic permeability value when compared to the corresponding membrane without nanoparticles (CA400-34) [18].

### 3.2. Molecular Weight Cut-Off

Table 3 presents the apparent organic solute rejections obtained for CA and CA/Ag membranes.

Following the procedure described in Section 2.4, the MWCO was determined for the membranes studied, these results are presented in Table 4.

For the membrane series CA400-22 and CA400-30, besides the expected increase in MWCO with the increased formamide content, an increase in MWCO with the increase in AgNP content is also observed. This behavior is not observed for the CA400-34 membranes series, as a decrease in MWCO is observed for the membrane with 0.4 wt% Ag; this may be attributed to the presence of AgNPs with larger dimensions associated with higher AgNP content and subsequent aggregation, as reported by Maheswari et al., that leads to higher rejections of solutes [7].

### 3.3. Zeta Potential and Surface Charge Density of CA Membranes

**Figure 2:**
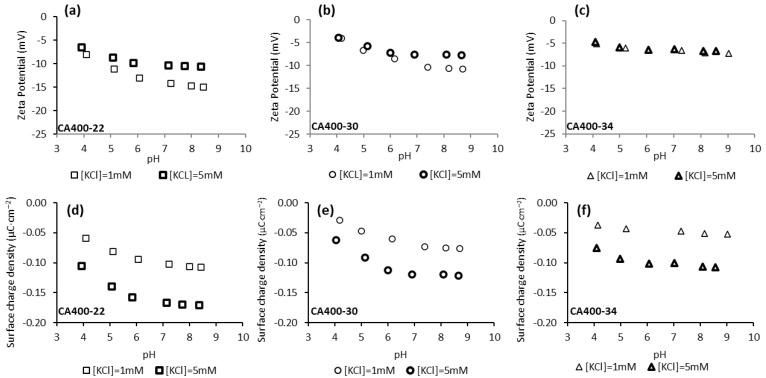
Surface-charge density at the shear plane (**a**–**c**) and zeta potential (**d**–**f**) of CA membranes surfaces as a function of pH and of solution ionic strength (potassium chloride solution 1 mM and 5 mM).

Figure 2 shows the zeta potential and surface charge density values obtained for CA400-22, CA400-30, and CA400-34 membranes in the experiments carried out with potassium chloride 1 mM and 5 mM and in the range of pH from 4 to 9. The results indicate that, in all cases, negative values were obtained for the CA membrane surfaces, and those results become more negative, in general, with the pH increase. Standard deviations of the zeta potential results are presented in supplementary information. According to the literature [35,36], the CA membranes have their isoelectric point (IEP) in the pH range of 3–4. At pH values above the IEP, the CA membranes are no longer electrostatically neutral, becoming negatively charged. As reported by Elimelech et al. [37], the negative charge acquired by the CA membranes cannot be explained by the dissociation of the acetyl and hydroxyl functional groups present in the polymeric structure, since these functional groups do not dissociate under the chemical conditions used in the experiments. Therefore, the explanation of the negative charge of CA membrane surfaces can be attributed to the adsorption of anions from the solution, Cl^−^ and OH^−^.

As can be observed in Figure 2, the zeta potential and surface-charge-density values become less negative with the increase in the membranes MWCO. Those results suggest that the zeta potential of membrane surface becomes less negative with the increase in membrane pore size, as previously reported in the literature [24,38,39]. Regarding the surface-charge density at the shear plane, obtained from the zeta-potential results by the Gouy–Chapman equation, Equation (5) [33], the results reveal that the denser membrane, CA400-22, exhibits a more negative surface-charge density compared to CA400-30 and CA400-34. This trend can be attributed to the higher polymer density at the surface of CA400-22, which allows for greater anion adsorption (e.g., Cl^−^ and OH^−^ ions).

Comparing the zeta-potential results obtained for the two electrolyte concentrations, the values obtained for the higher value (5 mM KCl) are, in general, less negative than those observed for the 1 mM (Figure 2). This is more pronounced for the CA400-22 membrane (Figure 2a) and the difference decreases for the CA400-30 and CA400-34 membranes. The decrease in zeta-potential absolute values under electrolyte solutions of higher ionic strength (5 mM) is related to the fact that the electric double layer is compressed, decreasing the Debye length thickness [40,41]. The Debye length thickness depends on the ionic strength of the electrolyte and can be determined through Equation (4), yielding the values of 9.62 and 4.30 nm for the electrolyte concentrations of 1 mM and 5 mM, respectively.

According to the literature, it is assumed that the electrical potential in the solution surrounding the surface decreases exponentially with the distance from the surface, so electrolytes with higher ionic strength and consequently lower Debye length will promote a more abrupt decrease in the potential between the surface and the diffuse layer, leading to absolute values of zeta potential that are smaller when compared to the values obtained with the electrolyte with lower ionic strength [32,40,41,42].

Through the results presented in Figure 2, it is observed that for the three CA membranes (CA400-22, CA400-30 and CA400-34) the surface-charge densities obtained with the more concentrated potassium chloride solution (5 mM) are more negative when compared with the results obtained with 1 mM. These results can be explained by the presence of a higher concentration of anion (Cl^−^) that will be adsorbed on the membrane surface, resulting in a more negative surface-charge density.

### 3.4. Zeta Potential and Surface Charge Density of CA/Ag Membranes

**Figure 3:**
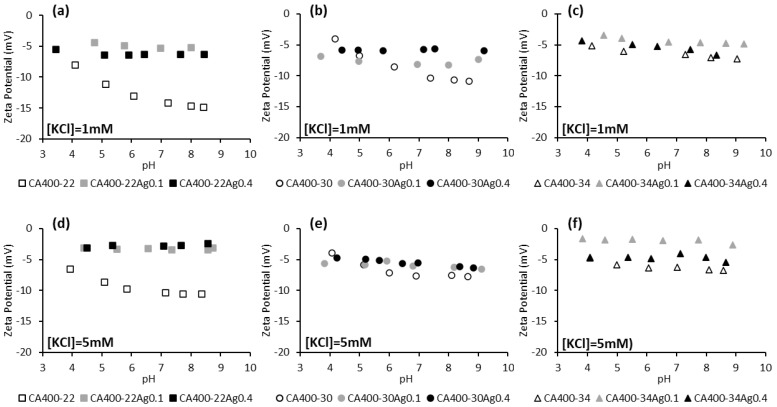
Zeta potential of CA and CA/Ag membranes surfaces as a function of pH, in a potassium chloride solution of 1 mM (**a**) CA400-22 series, (**b**) CA400-30 series, and (**c**) CA400-34 series; and 5 mM (**d**) CA400-22 series, (**e**) CA400-30 series, and (**f**) CA400-34 series.

Figure 3 displays the zeta potential of CA and CA/Ag membranes at two electrolyte concentrations (1 mM and 5 mM) in the pH range between 4 and 9. Standard deviations of the zeta potential results are presented in supplementary information.

The results reported in Figure 3 indicate, in general, that membranes with incorporated silver nanoparticles present less negative zeta-potential values when compared to the silver-free membranes. This behavior is more marked for the tightest membrane series, CA400-22. Some studies with membranes containing impregnated silver nanoparticles revealed a similar surface charge when compared with the silver-free membrane [19,43].

**Figure 4:**
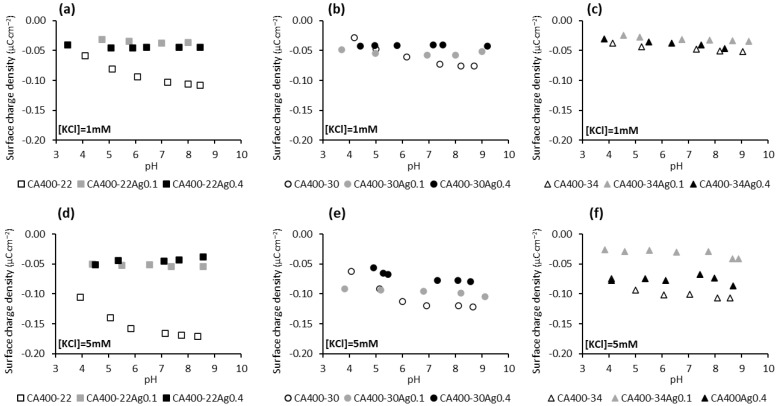
Surface-charge density at the shear plane of CA and CA/Ag membranes’ surfaces as a function of pH in a potassium chloride solution of 1 mM (**a**) CA400-22 series, (**b**) CA400-30 series, and (**c**) CA400-34 series; and 5 mM (**d**) CA400-22 series, (**e**) CA400-30 series, and (**f**) CA400-34 series.

The surface-charge density results presented in Figure 4 show that the surface of the membranes containing silver nanoparticles is less susceptible to adsorbing anions (Cl^−^ and OH^−^) from the solution, since with increasing concentrations of the electrolyte (and therefore a greater concentration of Cl^−^ ions), in general, a significant increase in the negative charge of the membrane is not observed, unlike what occurs with the silver-free membranes. Regarding the dependence of the surface-charge density of the silver-containing membranes with the pH of the solution (Figure 4), it is noted that an increase in the negative charge of the membrane is not observed with pH increase, suggesting that the anions (OH^−^ and Cl^−^) are not being adsorbed onto the membrane surface.

### 3.5. Bovine-Serum-Albumin Permeation Flux

**Figure 5:**
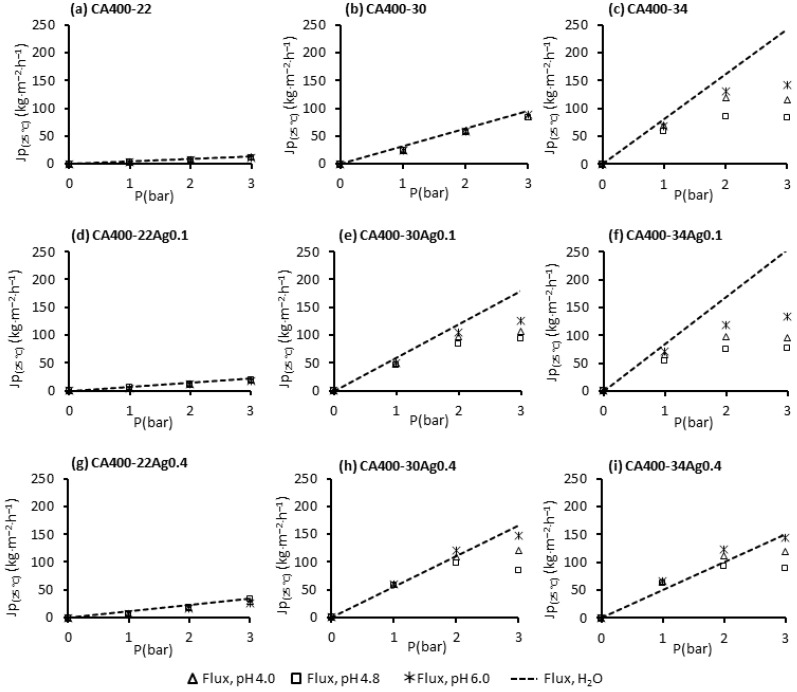
Permeate fluxes of pure water and BSA solutions (50 ppm at pH 4.0, 4.8 and 6.0) at 1, 2 and 3 bar, and with a feed flowrate of 180 L/h for the CA400-22 series: (**a**) 0 wt% Ag, (**d**) 0.1 wt% Ag and (**g**) 0.4 wt% Ag; CA400-30 series: (**b**) 0 wt% Ag, (**e**) 0.1 wt% Ag and (**h**) 0.4 wt% Ag; CA400-30 series: (**c**) 0 wt% Ag, (**f**) 0.1 wt% Ag and (**i**) 0.4 wt% Ag.

Figure 5 displays the permeate fluxes of pure water and BSA solutions measured at different transmembrane pressures (1, 2 and 3 bar) with a constant feed flowrate of 180 L/h operating in total recirculating mode (where both concentrate and permeate are recirculated to the feed tank). The BSA solutions were used at different pH values (4.0, 4.8 and 6.0) with a concentration of 50 ppm.

The results obtained for the CA400-22 (Figure 5a) and CA400-30 (Figure 5b) membranes show a linear relation between the permeate flux and the transmembrane pressure for the pure water and also for the BSA solutions in the pH range studied, although the BSA results are slightly lower. For the more porous membrane, CA400-34 (Figure 5c), the linearity between the flux and the transmembrane pressure is no longer observed; for transmembrane pressures above 2 bar, and the appearance of a flux plateau is observed, designated as limiting flux. Moreover, there is a pronounced flux decline when comparing it to the pure-water flux. A dominance of the concentration polarization is verified for the more porous membranes, and this obviously increases with the increase in the transmembrane pressure, as can be observed in Figure 5c.

The linearity between the transmembrane pressures and permeate fluxes is maintained for the denser membranes with incorporated AgNPs (CA400-22Ag0.1 (Figure 5d) and CA400-22Ag0.4 (Figure 5g), as was observed for the silver-free membrane CA400-22, which was the case for all pH values. This was not observed for the CA400-30 with 0.1 wt% Ag (Figure 5e) and 0.4 wt% Ag (Figure 5h), in which the linearity is not verified above 2 bar.

For the more porous membranes, in all cases, the appearance of a limiting flux above 2 bar is visible. It is also possible to see, in Figure 5f, that protein-solution fluxes are considerably lower than the pure-water fluxes, at all pH values studied, to transmembrane pressures above 2 bar. On the contrary, for CA400-34Ag0.4 membrane (Figure 5i), protein fluxes are close to the pure-water fluxes obtained for the transmembrane pressure between 1 and 3 bar. This is because for this membrane, the pure-water flux is much lower.

In general, the pH value that was conducive to lower permeate fluxes was observed at the IEP of BSA (pH 4.8). In this condition, the BSA molecules have no electric charge and the electrostatic repulsive force between the protein molecules is at its minimum, leading to higher agglomeration [44,45]. Proteins acquire electric charge when the pH of solution is different from the IEP. When the pH of protein solution is below its IEP (pH 4.0), the protein molecules acquire positive charge, and above IEP (pH 6.0) the protein is negatively charged.

The results obtained for BSA solutions with a pH value outside of its IEP, pH 4.0 and 6.0, evidenced that permeate fluxes were enhanced. At these conditions, electrostatic repulsions between the membrane surface and the protein molecules, and between protein molecules themselves, are present.

As referred to above, for pH 6.0, BSA molecules are negatively charged, and as presented in Section 3.3, the membrane surface is also negatively charged. When the membrane surface and the protein molecules have the same electric charges, an electrostatic repulsion between them may justify a lower fouling and the higher permeate fluxes observed at this pH value, for CA400-30 and CA400-34 membranes with 0 wt%, 0.1 wt%, and 0.4 wt% Ag. When the pH solution was adjusted to 4.0, the condition at which the membrane maintains the negative charge but protein molecules present positive charge, the permeate fluxes decrease in relation to the pH 6.0 condition, for CA400-30 and CA400-34 membranes with 0 wt%, 0.1 wt%, and 0.4 wt% Ag. This may be due to the different electric charges presented by the protein and the membrane, causing some electrostatic attraction; nevertheless, the permeate fluxes obtained in these conditions are higher than those observed for the pH 4.8 (IEP).

## 4. Conclusions

The zeta-potential measurements of CA membranes show that, for all the membranes prepared (CA400-22, CA400-30 and CA400-34), negative zeta-potential values were observed becoming more negative, in general, with the pH increase. In relation to the CA/Ag membranes, the zeta-potential values were less negative when compared to the silver-free membranes, and this is almost independent of both silver content and pH of solution, maintaining an almost constant value.

From the hydraulic permeability results, it was observed that for the denser membranes (CA400-22), the hydraulic permeability was enhanced with the incorporation of silver nanoparticles; for the structure CA400-30, an increase in permeability for the addition of 0.1 wt% Ag was observed, but the increase to 0.4 wt% Ag induces a decrease in the hydraulic permeability value. For the more porous structure (CA400-34), a slight increase was observed (5%) with the incorporation of 0.1 wt%, and a decrease was observed in cases with higher AgNP addition. The decrease in hydraulic permeability for higher silver nanoparticle content may be due to the presence of a higher amount of silver nanoparticles or with larger nanoparticles, as discussed previously, that could be trapped by the membrane matrix, blocking the pores.

The permeation experiments for organic solutes with different molecular weights evidenced that the CA/Ag membranes present lower apparent coefficient rejections when compared to the CA membranes. The exception is CA400-34Ag0.4, which presents apparent rejections similar to the corresponding silver-free membrane, CA400-34. Thus, the MWCO of CA/Ag membranes increase with the AgNPs’ incorporation (0.1 wt% and 0.4 wt% Ag), except for the addition of 0.4 wt% Ag in CA400-34 casting solution.

The permeation experiments using BSA shows that, in general, the more porous membrane displays a linear deviation between the permeate flux and the transmembrane pressure with the appearance of a limiting flux above 2 bar. The reduction in permeate fluxes can be caused by concentration polarization, the accumulation of materials within the pores by adsorption or pore blocking, and the accumulation of material at the membrane surface, forming a fouling layer. The influence of protein-solution pH was studied, and the results show that, in general, the pH value that is conducive to lower permeate fluxes is observed at IEP of BSA (pH 4.8); under this condition, the BSA molecules have no electric charge and the electrostatic repulsive force between the protein molecules is minimal.

## Figures and Tables

**Table 1:** Casting solutions compositions and film-casting conditions of the CA400 series membranes free of silver and with silver nanoparticles.

Membrane	CA400								
	22	22Ag0.1	22Ag0.4	30	30Ag0.1	30Ag0.4	34	34Ag0.1	34Ag0.4
**Casting solution (wt%)**									
Cellulose acetate	17.0	16.4	15.3	17.0	16.4	15.3	17.0	16.4	15.3
Formamide	22.0	21.2	19.8	30.0	29.0	27.0	34.0	32.8	30.6
Acetone	61.0	58.9	54.9	53.0	51.1	47.7	49.0	47.3	44.1
Silver nanoparticles									
Dispersion	-	3.4	9.6	-	3.4	9.6	-	3.4	9.6
Silver	-	0.1	0.4	-	0.1	0.4	-	0.1	0.4
**Casting Conditions**									
Temperature of solution (°C)	20–25								
Temperature of atmosphere (°C)	20–25								
Solvent evaporation time (min)	0.5								
Gelation medium	Ice-cold water (1–2 h)								

**Table 2:** Hydraulic permeability results obtained for the CA and CA/Ag membrane series [18].

	L_p_ (kg m^−2^ h^−1^ bar^−1^)		
	0 wt% Ag	0.1 wt% Ag	0.4 wt% Ag
CA400-22	3.50	7.05	11.16
CA400-30	32.05	66.85	59.72
CA400-34	80.88	84.48	59.10

**Table 3:** Apparent rejection coefficient to reference organic solutes obtained for the CA and CA/Ag membrane series.

	PEG (Da)						Dextran (Da)
	1000	3000	6000	8000	10,000	20,000	DT4000
CA400-22	57.6%	84.9%	95.9%	99.3%	-	-	-
CA400-22Ag0.1	11.6%	44.7%	88.9%	94.3%	-	-	-
CA400-22Ag0.4	2.4%	8.5%	55.4%	-	85.2%	95.5%	97.8%
CA400-30	-	42.4%	73.9%	-	89.0%	93.5%	95.1%
CA400-30Ag0.1	-	37.1%	66.3%	-	82.2%	92.9%	96.7%
CA400-30Ag0.4	4.6%	28.2%	56.1%	-	63.9%	74.6%	97.6%
CA400-34	-	24.5%	48.0%	-	63.3%	86.4%	95.5%
CA400-34Ag0.1	-	22.0%	27.9%	-	37.9%	44.6%	90.1%
CA400-34Ag0.4	1.8%	24.2%	46.0%	-	53.5%	67.0%	95.7%

**Table 4:** Molecular weight cut-off results obtained for the CA and CA/Ag membrane series [18].

	MWCO (kDa)		
	0 wt% Ag	0.1 wt% Ag	0.4 wt% Ag
CA400-22	4.17	6.86	15.35
CA400-30	8.32	17.58	26.52
CA400-34	31.43	41.05	31.96

## Data Availability

No new data were created or analyzed in this study. Data sharing is not applicable to this article.

## Supplementary Materials

The following supporting information can be downloaded at: https://www.mdpi.com/article/10.3390/membranes15020061/s1. Table S1: Zeta Potential average and standard deviation of CA membranes (CA400-22, CA400-30 and CA400-34) surface as a function of pH in a potassium chloride solution of 1 mM and 5 mM. Table S2: Zeta Potential average and standard deviation of CA/Ag membranes with 0.1% wt Ag (CA400-22Ag0.1, CA400-30Ag0.1 and CA400-34Ag0.1) surface as a function of pH in a potassium chloride solution of 1 mM and 5 mM. Table S3: Zeta Potential average and standard deviation of CA/Ag membranes with 0.4% wt Ag (CA400-22Ag0.4, CA400-30Ag0.4 and CA400-34Ag0.4) surface as a function of pH in a potassium chloride solution of 1 mM and 5 mM.
