# Supplementary material for: Surface-Charge Characterization of Nanocomposite Cellulose Acetate/Silver Membranes and BSA Permeation Performance
Source: Membranes (Basel). 2025 Feb 11;15(2):61. doi: 10.3390/membranes15020061 (PMC11857461; doi:10.3390/membranes15020061)
Supplement: Supplementary file 1 [file membranes-15-00061-s001.zip › membranes-3405191-supplementary.pdf]

membranes

MDPI

Supplementary Information

Table S1 – Zeta Potential average and standard deviation of CA membranes (CA400-22, CA400-30 and CA400-34) surface as a function of pH in a potassium chloride solution of 1mM and 5mM.

	[KCl]=1mM			[KCl]=5mM		
	**pH**	**Average**	**STD**	**pH**	**Average**	**STD**
**CA400-22**	4.098	-8.071	0.171	3.927	-6.537	0.168
	5.127	-11.148	0.305	5.079	-8.694	0.226
	6.071	-13.056	0.385	5.850	-9.813	0.339
	7.225	-14.223	0.428	7.142	-10.359	0.432
	7.998	-14.728	0.417	7.740	-10.540	0.519
	8.436	-14.922	0.394	8.360	-10.606	0.816
**CA400-30**	4.182	-3.733	0.837	4.061	-3.644	0.552
	4.996	-6.165	1.376	5.133	-5.331	0.356
	6.166	-7.839	1.316	5.999	-6.589	0.567
	7.397	-9.501	1.319	6.907	-7.011	0.279
	8.195	-9.818	1.198	8.100	-6.984	0.459
	8.699	-9.952	1.564	8.655	-7.142	0.169
**CA400-34**	4.138	-5.109	0.368	4.092	-4.639	0.346
	5.221	-6.001	0.315	4.991	-5.857	0.673
	-	-	-	6.065	-6.405	0.491
	7.287	-6.530	0.372	7.028	-6.300	0.434
	8.166	-7.096	0.769	8.089	-6.683	1.037
	9.035	-7.243	0.474	8.566	-6.710	0.985

Table S2 – Zeta Potential average and standard deviation of CA/Ag membranes with 0.1% wt Ag (CA400-22Ag0.1, CA400-30Ag0.1 and CA400-34Ag0.1) surface as a function of pH in a potassium chloride solution of 1mM and 5mM.

	**[KCl]=1mM**			**[KCl]=5mM**		
	**pH**	**Average**	**STD**	**pH**	**Average**	**STD**
**CA400-22Ag0.1**	4.745	-4.814	0.727	4.399	-3.132	0.731
	5.766	-5.198	0.685	5.510	-3.317	0.241
	6.985	-5.488	1.154	6.554	-3.265	0.715
	7.996	-5.269	0.437	7.361	-3.460	0.136
	9.847	-5.710	0.705	8.564	-3.474	0.125
**CA400-30Ag0.1**	3.695	-6.841	1.237	3.812	-5.620	1.257
	4.977	-7.651	0.993	5.185	-5.868	0.740
	-	-	-	5.922	-5.294	1.706
	6.937	-8.137	1.145	6.797	-6.026	1.431
	7.987	-8.226	1.364	8.191	-6.240	1.421
	8.995	-7.348	0.288	9.104	-6.584	1.386
**CA400-34Ag0.1**	4.548	-3.414	0.365	3.836	-1.613	1.686
	5.161	-3.893	0.190	4.578	-1.854	1.315
	6.738	-4.505	0.198	5.513	-1.723	1.061
	7.797	-4.671	0.486	6.543	-1.894	0.211
	8.657	-4.748	0.706	7.737	-1.818	0.686
	9.267	-4.849	0.756	8.886	-2.637	2.064

Table S3 – Zeta Potential average and standard deviation of CA/Ag membranes with 0.4% wt Ag (CA400-22Ag0.4, CA400-30Ag0.4 and CA400-34Ag0.4) surface as a function of pH in a potassium chloride solution of 1mM and 5mM.

	**[KCl]=1mM**			**[KCl]=5mM**		
	**pH**	**Average**	**STD**	**pH**	**Average**	**STD**
**CA400-22Ag0.4**	3.444	-5.584	0.551	4.496	-3.162	0.901
	5.079	-6.428	0.376	5.363	-2.781	0.715
	5.897	-6.427	0.242	-	-	-
	6.431	-6.325	0.344	7.085	-2.848	0.203
	7.650	-6.382	0.891	7.661	-2.740	0.373
	8.444	-6.303	0.726	8.563	-2.416	1.342
**CA400-30Ag0.4**	4.412	-5.802	2.652	4.240	-4.783	2.715
	4.960	-5.890	0.973	5.210	-4.912	1.027
	5.796	-5.903	0.442	5.673	-5.106	0.624
	-	-	-	6.433	-5.627	0.774
	7.159	-5.790	0.139	6.963	-5.534	1.216
	7.528	-5.695	0.397	8.370	-4.461	-6.173
	9.198	-5.997	0.181	8.845	-6.318	1.718
**CA400-34Ag0.4**	3.817	-4.301	0.544	4.083	-4.791	0.952
	5.506	-4.974	0.709	5.367	-4.674	0.913
	6.356	-5.228	1.084	6.132	-4.850	0.532
	7.472	-5.758	0.547	7.117	-4.008	1.220
	-	-	-	7.979	-4.646	1.795
	8.347	-6.623	1.477	8.663	-5.492	1.368
